# Editorial: Cardiac energetic efficiency and cardiometabolic diseases

**DOI:** 10.3389/fcvm.2023.1352798

**Published:** 2023-12-21

**Authors:** Teresa Vanessa Fiorentino, Francesca Cinti, Ram Jagannathan

**Affiliations:** ^1^ Department of Medical and Surgical Sciences, University “Magna Graecia” of Catanzaro, Catanzaro, Italy; ^2^ Centro Malattie Endocrine e Metaboliche, Dipartimento di Scienze Mediche e Chirurgiche, Fondazione Policlinico Universitario A. Gemelli IRCCS and Università Cattolica del Sacro Cuore, Roma, Italy; ^3^ Division of Hospital Medicine, Department of Medicine, Emory University School of Medicine, Atlanta, GA, United States

**Keywords:** myocardial efficiency, cardiovascular disease, cardiac energetics, cardiac metabolism, cardiac performance

**Editorial on the Research Topic** Cardiac energetic efficiency and cardiometabolic diseases

Increasing evidence have suggested that a compromised myocardial energetics is implicated in the pathogenesis of cardiovascular disease including ischemic cardiomyopathy, left ventricular hypertrophy and heart failure. The present Research Topic collects some of the last investigations evaluating the mechanisms underlying the link between an altered myocardial energetic efficiency and cardiovascular diseases, and the utility of pharmacological and non-pharmacological approaches targeting cardiac energetic metabolism to counteract cardiovascular disease progression.

The compilation starts with a cross-sectional study conducted by Liu et al. evaluating the association between coronary microvascular dysfunction and altered heart energetic efficiency and contractility. The Authors report that subjects with coronary microvascular dysfunction, defined as a reduced coronary flow reserve, exhibit a reduction in global work index, global contractive work, and global work efficiency and a higher global waste work, suggesting that coronary microvascular dysfunction may affect myocardial energetic efficiency and contractility. Additionally, the Authors also demonstrate that a reduced myocardial global work and efficiency may be a predictor of coronary microvascular dysfunction with a good diagnostic capacity, thus indicating that a compromised cardiac mechano-energetic efficiency may be a diagnostic tool for early identification of individuals with coronary artery disease.

Energetic metabolism of heat is a dynamic process depending of the availability of oxygen and several metabolic substrates such as glucose, fatty acids, ketone bodies and ammino acids. In order to gain insight into the metabolic alterations associated to a worse prognosis in subjects with coronary artery diseases, Na et al. conducted an observational study on 5,935 patients admitted to the cardiovascular department of Guang'anmen Hospital who were diagnosed with coronary heart disease and subdivided into two groups according to the occurrence of adverse myocardial events (MACEs) during the hospitalization. The Authors found that age, blood glucose, fatty acid, albumin, and ApoA1 levels at admission were associated with an increased risk of MACEs during the hospitalization. Decreased levels of blood glucose were associated with an increased risk of MACEs, whereas higher levels of glucose, making this energetic substrate more easily available, were protective. Higher levels of fatty acids and decreased values of Apo A1 were associated with an increased risk of MACEs, probably due to the accumulation of lipids in conditions of oxygen deprivation and their toxic effects on the heart. Additionally, decreased albumin concentrations were found to predict MACEs occurrence, indicating that a decreased availability of ammino acids for energetic supply may aggravates cardiac damage in conditions of myocardial hypoperfusion. The results of this study not only provide evidence that several energetic substrate changes occur in the heart of subjects with coronary artery disease but also demonstrate that metabolic alterations may predict MACEs, thus representing a tool for a better cardiovascular risk stratification and a potential therapeutic target.

Amongst chemical compound able to positively modulate energetic metabolism, polyphenols have attracted considerable attention. In their review Hedayati et al. describe the various benefic properties of polyphenols, including anti-inflammatory, antioxidant, antiapoptotic, and antiatherogenic effects thus suggesting that polyphenols may be promising adjutant approaches in the treatment of cardiovascular disease and heart failure.

The collection also includes preclinical studies with translational impact, providing new insights and interesting tools for clinical research on this topic field.

Raposo et al. report that intracoronary transfer of xenogeneic Human umbilical cord matrix-mesenchymal stromal cells (hUCM-MSC), shortly after reperfusion, improved left-ventricular systolic function. By using a swine randomized, sham- and placebo-controlled blinded trial, they demonstrated that improvement in mechanical performance may be depended to a favorable modification of myocardial interstitial fibrosis and downregulation of genes related to matrix remodeling, whereas the reduction of morphological infarct size seems to not be involved.

The treatment to protect heart function in post-myocardial infarction heart failure (post-MI HF) is still on debate. Tao et al. addressed this topic by testing the effect of the early administration of the SGLT-2i Dapagliflozin (DAPA) in rats post-MI HF compared to the combination of DAPA with sacubitril-valsartan (S/V) in different orders. They conclude that the most effective treatment strategy for rats with post-MI HF was the administration of DAPA during the first 2 weeks, followed by the addition of sacubitril-valsartan to DAPA later.

Last, the collection ends with a study to test the efficacy of supplementation of cardioplegia with sildenafil in a piglet model of cardiopulmonary bypass and arrest, using both cold and warm cardioplegia protocols. Previous investigations into whether sildenafil has a cardiac inotropic effect have proved controversial. Several human studies have found no evidence of an inotropic effect of sildenafil, either *in vivo* or *in vitro*, while others have reported that sildenafil administration is associated with increases in cardiac index. This study demonstrated that supplementation of cardioplegia with sildenafil has beneficial metabolic effects. Since cardioprotection for paediatric patients is currently not as effective as in adult patients and for a long time has been described as inadequate, these findings may represent an exciting step towards improving cardioprotection for this highly vulnerable group of patients.

In conclusion, the article Collection provides new insights for the role of myocardial energetic efficiency in the pathogenesis of cardiovascular diseases and explores the mechanisms affecting cardiac mechano-energetic performance providing potential solutions for clinical practice.

